# Recurrent Gastropericardial Fistula: A Case Report

**DOI:** 10.7759/cureus.82507

**Published:** 2025-04-18

**Authors:** Steven Pong, Herbert Downton Ramos, Justin Van Backer

**Affiliations:** 1 Department of Surgery, Frank H. Netter MD School of Medicine, Quinnipiac University, North Haven, USA; 2 Department of General Surgery, University of Connecticut School of Medicine, Farmington, USA; 3 Division of General Thoracic Surgery, Hartford HealthCare, Hartford, USA

**Keywords:** gastropericardial fistula, gastropleural fistula, intrathoracic gastric fistula, pneumopericardium, upper gastrointestinal surgery

## Abstract

Gastropericardial fistula (GPF) is a rare but serious complication that can develop years after upper gastrointestinal surgeries. We present the third documented case of recurrent GPF in a 77-year-old male with a complex surgical history, including hiatal hernia repair and multiple thoracic procedures. The patient exhibited nonspecific symptoms, including chest pain, vomiting, and signs of sepsis. Diagnosis was confirmed through CT and esophagogastroduodenoscopy. Treatment involved several surgical interventions: creation of a pericardial window, takedown of the GPF, and placement of an omental flap. This case highlights the importance of early diagnosis, prompt and aggressive surgical management, and diligent postoperative care in addressing GPF.

## Introduction

Gastropericardial fistula (GPF) is a rare and severe complication defined by an abnormal connection between the stomach and the pericardium. With only about 65 cases reported in the literature, GPF has been linked to various etiologies, including prior surgery, malignancy, gastric ulcer, trauma, NSAID use, and Zollinger-Ellison syndrome [1,2]. Among these, the most common causes are surgeries involving the upper gastrointestinal tract, such as hiatal hernia repairs, Nissen fundoplication, bariatric procedures, esophagectomy, and surgeries for esophageal cancer [1,2].

GPFs present significant diagnostic and therapeutic challenges due to their unpredictable clinical manifestations. A review of published cases reported a mortality rate of up to 69% prior to 2000, which declined to 11% in cases reported after 2000 - likely reflecting advancements in imaging techniques and surgical management [2]. On average, the interval between surgery and the development of a fistula is approximately seven years. Clinical presentations vary widely, from vague chest and epigastric pain to life-threatening sepsis and cardiac tamponade. The most common findings are sepsis and electrocardiographic evidence of pericarditis. Diagnosis typically relies on CT and endoscopy, with standard management involving surgical resection and pericardial drainage.

Here, we report a rare case of recurrent GPF in a patient with a complex surgical history, including hiatal hernia repair with fundoplication, right video-assisted thoracoscopic surgery (VATS) with mediastinal washout, and pericardial window creation.

## Case presentation

A 77-year-old male with a medical history of hypertension, hyperlipidemia, paroxysmal atrial fibrillation on apixaban 5 mg twice daily (Eliquis^®^, Bristol Myers Squibb, Princeton, NJ, USA and Pfizer, New York, NY, USA), ulcerative colitis (UC), and sick sinus syndrome status post permanent pacemaker placement experienced a recurrent GPF stemming from a complex surgical history that began with a hiatal hernia repair in 2011.

**Figure 1:**
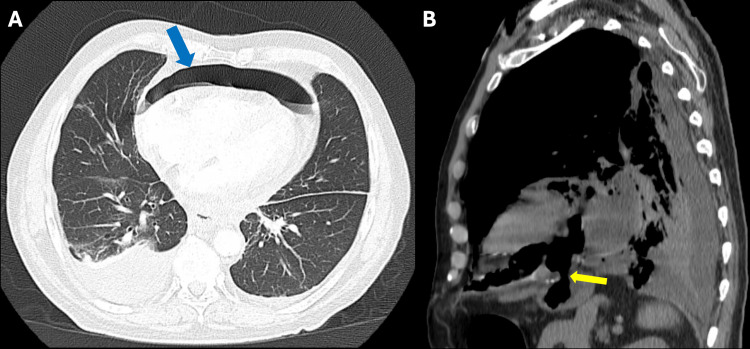
Chest CT scans from 2017 (A) CT scan on the day of presentation showing clear pneumopericardium (blue arrow). (B) CT scan taken two days later, following the patient’s acute onset of shoulder pain and shortness of breath. The yellow arrow indicates the location of the fistula.

**Figure 2:**
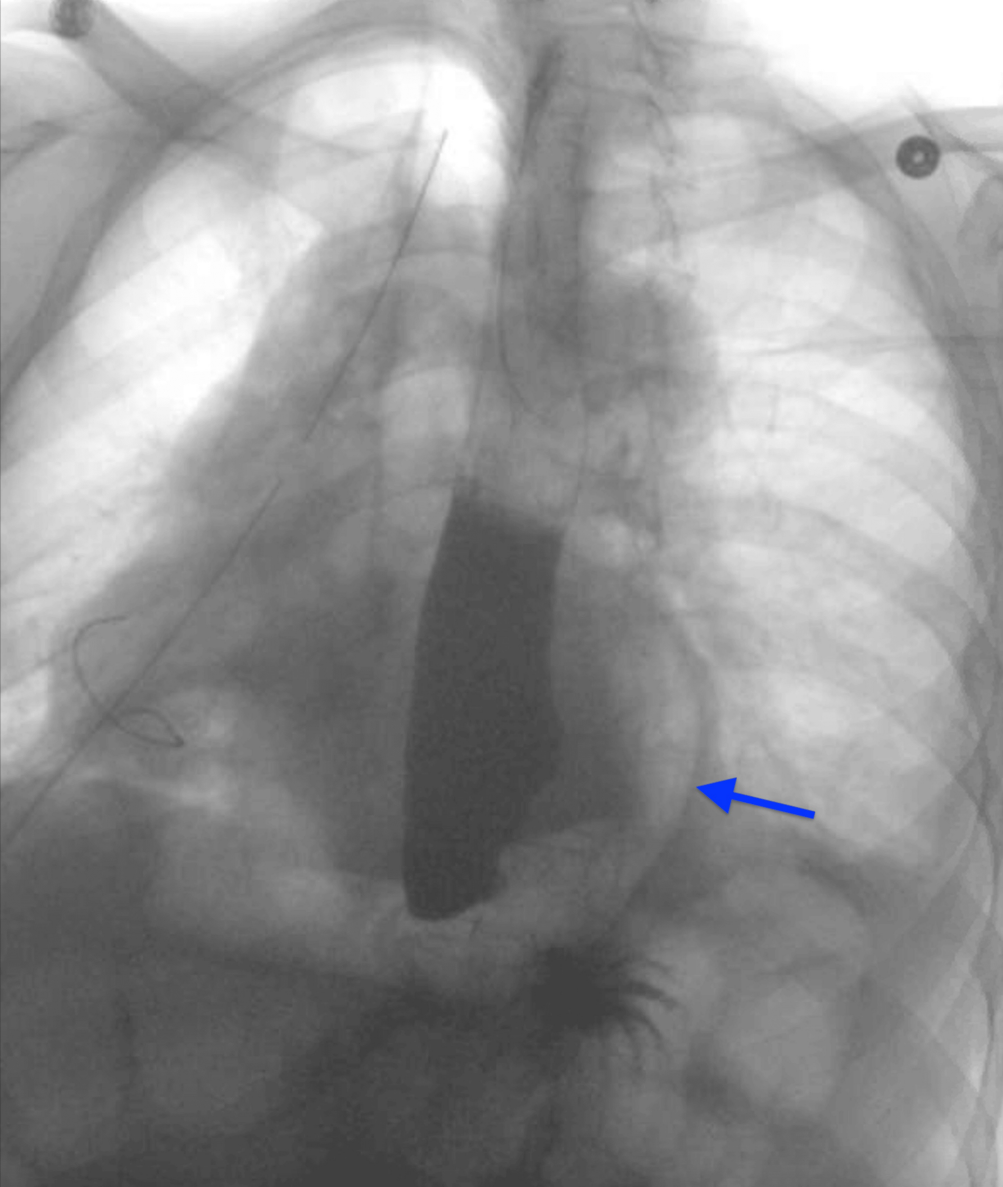
Initial esophagram from 2017 The initial barium swallow study shows a small amount of contrast extending superiorly from the proximal stomach, raising suspicion for extravasation into the pericardium (arrow).

In 2016, he presented with pleuritic chest pain, and a CTA revealed a large left hydropneumothorax, which was managed with chest tube placement. In 2017, he returned with chest pain and emesis; a CT scan revealed pneumomediastinum and pneumopericardium (Figure 1). This prompted an esophagogastroduodenoscopy (EGD), right VATS with chest and mediastinal washout, and pericardial window creation. An initial esophagram confirmed the first GPF, which was managed with a partial wedge gastrectomy, stapled resection of the fistula, and GJ tube placement (Figure 2).

**Figure 3:**
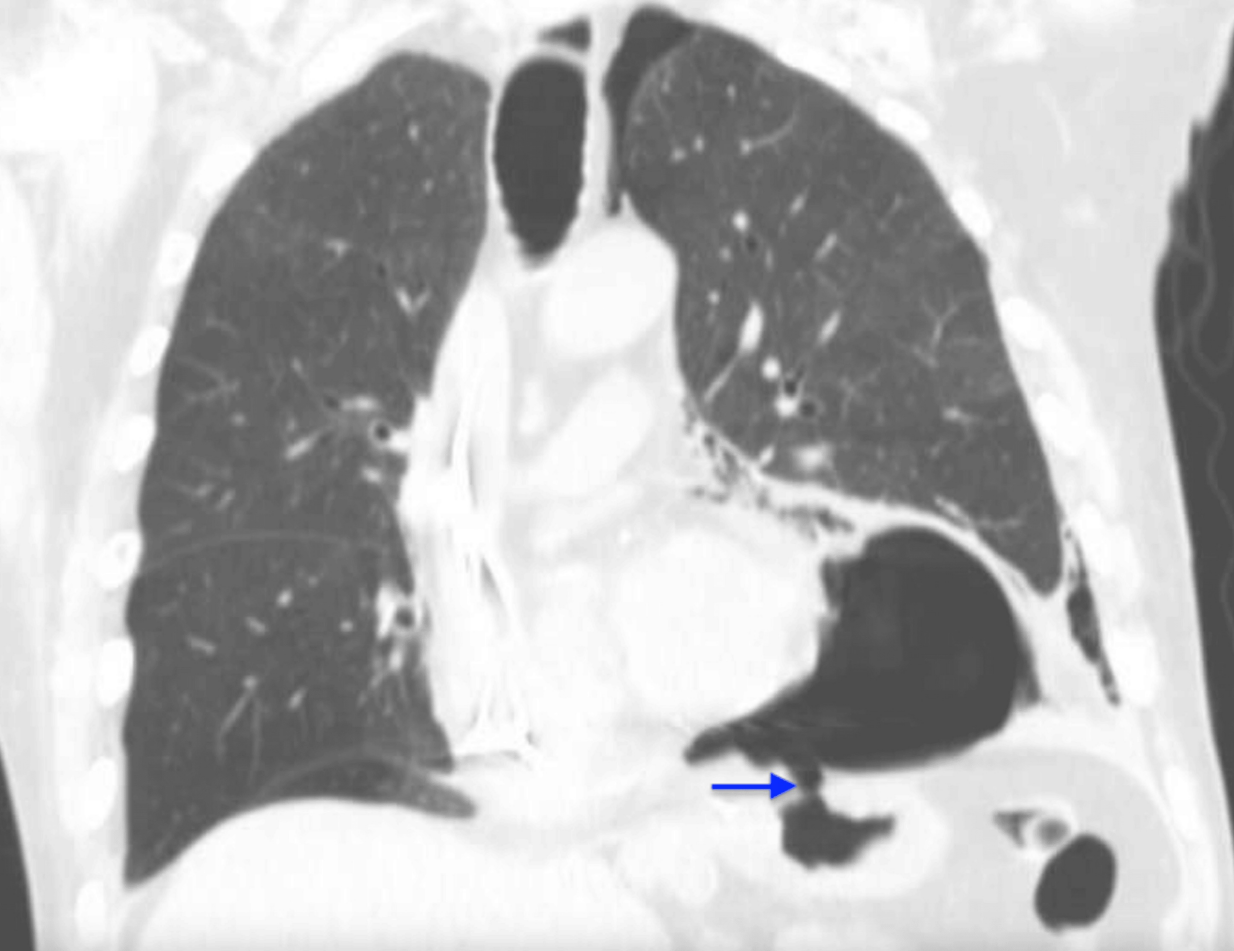
Coronal chest CT scan from 2019 CT scan demonstrates pneumopericardium and a second GPF (arrow). GPF, gastropericardial fistula

**Figure 4:**
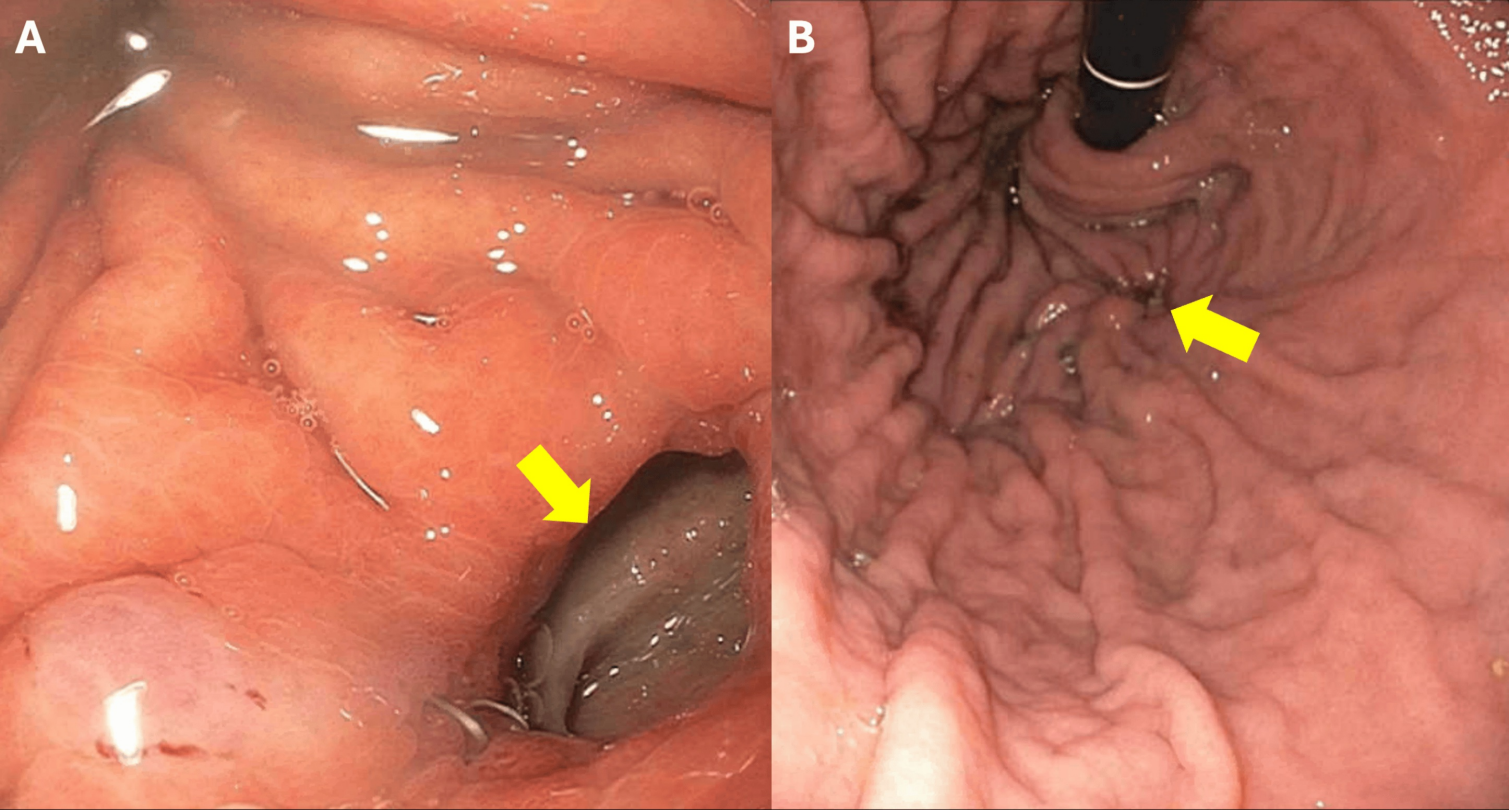
EGD from 2019 (A) EGD showing the second recurrent GPF (arrow). (B) Follow-up EGD performed three months postoperatively, showing the gastric fundus at the site of fistula repair. The arrow indicates intact clips with no evidence of recurrent fistula. EGD, esophagogastroduodenoscopy

In 2018, the patient underwent an urgent Graham patch repair for a perforated gastric ulcer. In 2019, he had a laparoscopic subxiphoid incisional hernia repair with mesh placement but later presented with chest and abdominal pain. Imaging revealed recurrent pneumomediastinum and pneumopericardium (Figure 3), prompting an EGD (Figure 4A), followed by left VATS decortication and pericardial window creation.

The second recurrent fistula was endoscopically clipped, and the patient was discharged after a favorable recovery. Later in 2019, a repeat EGD confirmed that the clips were in place, with no recurrence of the fistula observed (Figure 4B).

**Figure 5:**
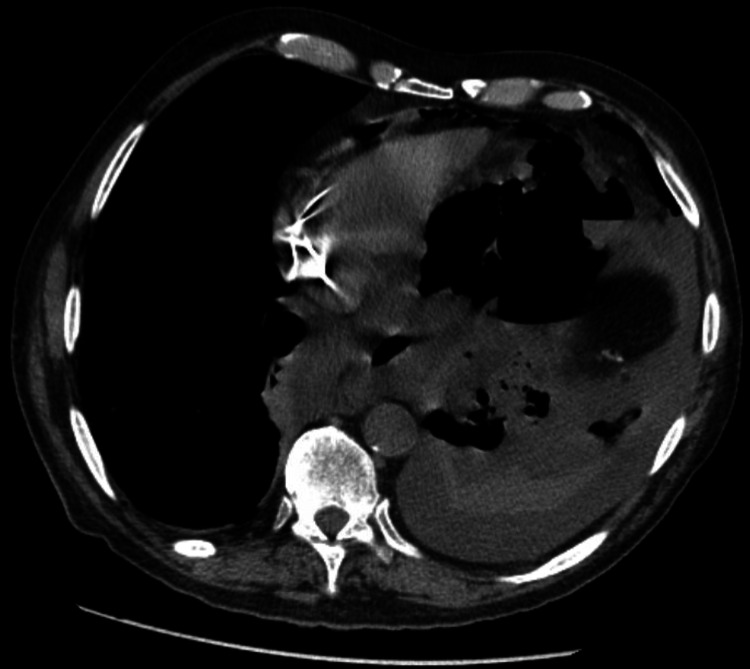
Axial chest CT scan from 2024 CT scan reveals a third gastropleural fistula accompanied by a large fluid collection in the left hemithorax.

**Figure 6:**
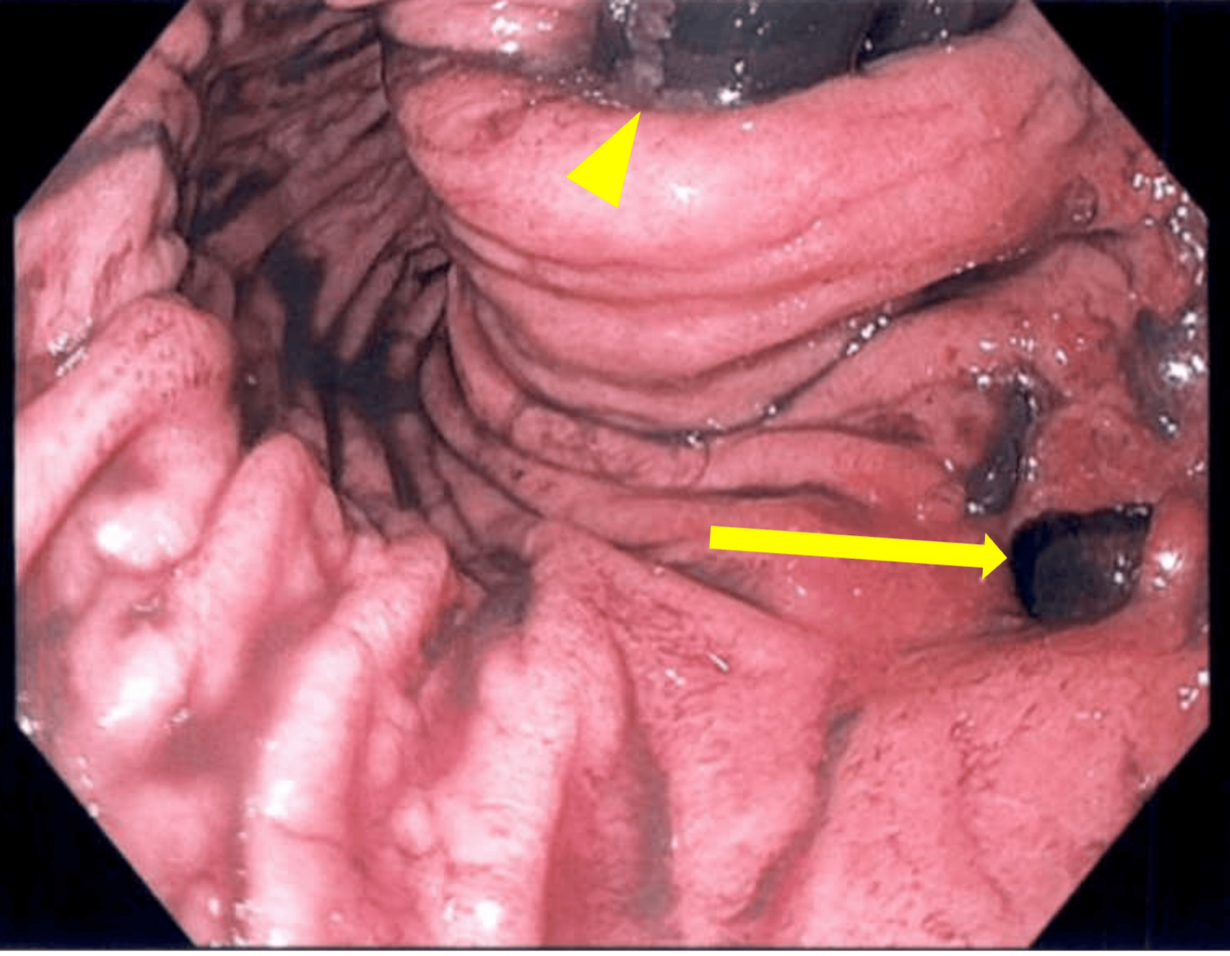
EGD of recurrent GPF from 2024 EGD shows the third recurrent fistula (arrow) with an intact fundoplication (triangle). EGD, esophagogastroduodenoscopy; GPF, gastropericardial fistula

In 2024, the patient experienced hypoxia, shortness of breath, and signs of sepsis. A CT scan revealed a third gastropleural fistula with a large fluid collection in the left hemithorax (Figure 5). The patient was taken to the operating room for bronchoscopy and EGD, which identified a third 1-cm gastric fistula (Figure 6). This was managed with left VATS decortication, wedge resection, GPF takedown, and omental flap placement through exploratory laparotomy, along with GJ tube placement. The patient had a favorable postoperative recovery and was treated with antibiotics for empyema.

## Discussion

Our case illustrates the recurrent nature of GPF in a patient with a complex surgical history. The key to managing GPF is early recognition and surgical intervention. Diagnostic imaging plays a critical role, with chest radiographs revealing pneumopericardium and thickened pericardium, while CT scans can identify air-fluid levels due to hydropneumopericardium and teardrop-shaped gas pointing toward the heart [3]. EGD is essential for directly visualizing the fistulous tract, with a diagnostic success rate of 86% in reported cases.

Surgical management typically includes resection of the fistula, pericardial space drainage, and primary repair of the gastric defect. In more complex or recurrent cases, additional procedures such as omental or muscle flap interposition are recommended to reinforce the repair and reduce the risk of recurrence [4]. A systematic review and pooled analysis of 25 studies, encompassing 76 patients with post-bariatric intrathoracic gastric fistula (ITGF), including GPF, found that surgical treatment combined with endoscopic techniques resulted in complete resolution in 71.25% of cases [5]. Our patient’s case underscores the chronic and recurrent nature of GPF, which can present in various forms of ITGF, including as a gastropleural fistula.

Although UC is not typically associated with fistula formation in the same way as Crohn’s disease (CD), it remains clinically relevant in this case. Endoscopic findings from 2015 revealed “burnt-out colitis” with pseudopolyps, and biopsies showed mild chronic quiescent colitis with no evidence of dysplasia or active inflammation. Additionally, no gross evidence of active inflammation was observed during surgery. Our patient’s UC was reportedly well-controlled on mesalamine 4.8 mg daily, suggesting a regimen aimed at maintaining mucosal remission, with stable disease and no escalation to immunosuppressive therapy. This is notable because immunosuppression and mucosal ulceration may increase the risk of fistulizing complications [6]. A recent Mendelian randomization study supports a causal link between CD and fistula, particularly in the anal and rectal regions, but found no causal association between UC and fistula formation after adjusting for confounding factors using multivariable MR analysis [7]. This suggests that while UC may not independently predispose patients to fistula formation, an upper-GI surgical history or additional risk factors, such as prior ulceration, could contribute. However, a role for subclinical inflammation in impaired tissue healing, even in histologically quiescent UC, cannot be fully excluded [8]. In our case, the recurrent GPF is likely multifactorial, with contributions from prior foregut surgery, possible subclinical inflammation, and impaired healing rather than UC alone.

To our knowledge, this is only the third documented case of recurrent GPF (Table 1). The first case, reported by Servais et al. (2012), involved a 24-year-old man who developed a recurrent GPF over 20 years after undergoing gastric conduit reconstruction for congenital esophageal atresia. After an initial presentation and drainage for a GPF without resection, the patient re-presented five years later with pyopneumopericardium. Definitive repair required sternotomy, fistula excision, and reinforcement with a rectus abdominis flap, resulting in an uneventful recovery [9]. The second case, described by Granchi et al. (2016), involved a 42-year-old male who developed a gastrocardiac fistula following a delayed presentation of recurrent GPF after Nissen fundoplication. Despite two previous repairs - including a pericardial window, gastric repair, and omental interposition - the patient re-presented in extremis with gastrointestinal bleeding. Operative exploration revealed a fistula from the gastric fundus into the left ventricle, which was successfully repaired via sternotomy and pledgeted sutures [10].

**Table 1:** Clinical summary of three reported cases of recurrent GPFs GPF, gastropericardial fistula; PW, pericardial window; VATS, video-assisted thoracoscopic surgery

Case	Author (year)	Patient demographics	Initial presentation	Recurrence timeline	Surgical history	Management approach of recurrent GPF	Outcome
1	Servais et al. (2012) [9]	24-year-old male	Chest pain, chills, shortness of breath	Initial GPF at age 19, recurrence five years after initial drainage	Repair of congenital esophageal atresia with gastric conduit reconstruction, two reoperations, thoracotomy/PW	Sternotomy, fistula excision, rectus abdominis flap interposition	Successful repair, uneventful recovery
2	Granchi et al. (2016) [10]	42-year-old male	Melena, hypotension, tachycardia, malaise	Initial GPF at age 46, four years after Nissen fundoplication; second recurrence after 12 months, third after 15 months	Nissen fundoplication (2009), pericardial window, fistula takedown, primary suture repair with omental patch interposition, revision of Nissen fundoplication	Emergent endoscopy, gastrotomy, sternotomy, primary repair of fundus defect, myocardial repair with pledgeted sutures	Successful repair, postoperative empiric antibiotics with fungal coverage
3	Our study	77-year-old male	Chest pain, emesis, hypoxia, sepsis	First GPF in 2017, six years after hernia repair; second in 2019; third in 2024	Fundoplication (2011), wedge gastrectomy, mesh hernia repair, multiple VATS/pericardial windows, Graham patch repair	Endoscopic clipping, multiple VATS decortications, final takedown with omental flap	Successful repair, empyema treated with empiric antibiotics

The rarity of GPF presents significant challenges in developing standardized treatment protocols. Our case contributes to the growing recognition that recurrent GPF can occur long after initial surgical repair, particularly in patients with a complex surgical history. It emphasizes the importance of complete fistula excision and the consideration of flap interposition during the initial management to prevent recurrence.

Given its low incidence, with this being the third documented case of recurrent GPF, prospective randomized trials are unlikely to be feasible. However, this case highlights the need for heightened awareness and a high index of suspicion in patients presenting with recurrent symptoms following upper gastrointestinal surgery. Given these challenges, multicenter registries or pooled retrospective case series could serve as valuable tools for better characterizing clinical presentation patterns, identifying risk factors, and developing effective management strategies for this life-threatening condition.

## Conclusions

GPF is a rare but serious complication of upper gastrointestinal surgeries. Early recognition and definitive diagnosis are best achieved through CT imaging and EGD follow-up. Prompt surgical intervention and diligent postoperative care are crucial for improving patient outcomes. While standardized protocols for managing GPF remain elusive due to its rarity, future efforts could benefit from aggregating data across institutions to better define diagnostic and therapeutic best practices. Additionally, exploring minimally invasive surgical techniques and novel endoscopic interventions may provide further options for managing this challenging condition.
